# A machine learning-driven prognostic model based on peripheral blood lymphocyte subsets in osteosarcoma

**DOI:** 10.3389/fimmu.2026.1733518

**Published:** 2026-01-28

**Authors:** Longqing Li, Jinlei Liu, Songtao Pang, Yuan Zhao, Yimeng Wang, Jia Wen, Yongkui Liu, Yi Zhang, Yan Zhang, Jiazhen Li, Nan Zhou, Xinchang Lu

**Affiliations:** 1 Department of Orthopedics, The First Affiliated Hospital of Zhengzhou University, Zhengzhou, China; 2 Department of Obstetrics and Gynecology, The First Affiliated Hospital of Zhengzhou University, Zhengzhou, China

**Keywords:** machine learning, osteosarcoma, peripheral blood lymphocyte subsets, prognostic model, risk stratification

## Abstract

**Background:**

The prognosis of osteosarcoma (OS) remains heterogeneous, and the prognostic value of peripheral blood lymphocyte subsets, analyzed through machine learning (ML), is not fully explored. This study aimed to develop an ML-based prognostic model using lymphocyte subset data to improve risk stratification for OS patients.

**Methods:**

We retrospectively analyzed data from 65 high-grade OS patients. Peripheral blood lymphocyte subsets were quantified by flow cytometry prior to treatment. Seven algorithms, including stepwise Cox, LASSO, and five ML models (RSF, GBM, XGBoost, SVM, KNN), were employed to construct prognostic models. Model performance was evaluated using the C-index, AUC, and validated via bootstrap and cross-validation.

**Results:**

The Gradient Boosting Machine (GBM) algorithm yielded the optimal two-variable model, incorporating CD3^-^CD56^+^ NK cells and CD8^+^HLA-DR^+^ activated cytotoxic T cells (AUC = 0.959). The resulting gbm_risk_score was an independent prognostic factor (HR = 14.516, P = 0.012) and effectively stratified patients into significantly divergent survival groups (P<0.001). Importantly, the gbm_risk_score demonstrated superior predictive performance for 3-year OS compared to traditional inflammatory indices, neutrophil-to-lymphocyte ratio (NLR) and platelet-to-lymphocyte ratio (PLR). A nomogram integrating the GBM risk group and primary metastasis status demonstrated excellent predictive accuracy (C-index: 0.883) and clinical utility, successfully identifying a high-risk subgroup among initially non-metastatic patients.

**Conclusion:**

We developed and validated a robust ML-driven prognostic model based on peripheral blood lymphocyte subsets. This model, demonstrating superior prognostic value over conventional inflammatory markers, provides a novel and practical tool for personalized risk assessment in OS, potentially guiding more tailored treatment strategies.

## 1 Introduction

Osteosarcoma (OS) is a primary malignant bone tumor characterized by the production of osteoid tissue, most commonly occurring in the metaphysis of long bones in adolescents aged 10–20 years (1). Prior to the 1970s, treatment relied primarily on surgery alone, with poor outcomes; up to 80% of patients ultimately died from pulmonary metastases, and the 5-year overall survival (OS) rate was less than 20%. The landscape changed significantly by the 1980s. A comprehensive treatment protocol incorporating preoperative chemotherapy, surgery, and postoperative chemotherapy was established as the standard of care. This multimodal approach increased the 5-year OS rate to over 60% for patients without metastases at initial diagnosis (2, 3). However, the management of OS continues to pose significant challenges. Approximately 15% of patients present with metastases at diagnosis, conferring a very poor prognosis. Furthermore, about 30% of patients without metastases initially may develop metastases during treatment, leading to a substantial decline in survival (4). While novel therapies, such as targeted treatments and immunotherapy, have been applied for OS in recent years, their efficacy in improving outcomes for patients with metastatic disease remains limited (5, 6). Consequently, the Children’s Oncology Group (COG) suggests that the early identification of high-risk patients and the development of individualized treatment plans may represent another crucial pathway for improving prognosis (7).

Lymphocytes, as core components of the immune system, play an increasingly recognized role in tumor initiation, progression, and control. Multiple studies have shown that the presence of tumor-infiltrating lymphocytes (TILs), particularly CD8+ T cells, in tumor tissue is generally associated with a more favorable prognosis (8, 9). Similarly, biomarkers derived from peripheral blood lymphocyte counts, such as the neutrophil-to-lymphocyte ratio (NLR), have been established as independent prognostic factors for various solid tumors, including OS, with a lower pre-treatment NLR often correlating with better survival outcomes (10–12). Advances in lymphocyte research have revealed the complex and often opposing functions of different lymphocyte subsets in tumor progression. For instance, while CD8+ T cells can directly kill tumor cells, regulatory T cells (Tregs) may suppress immune responses and potentially promote tumor growth (13, 14). However, conventional blood tests typically report only the total lymphocyte count and cannot differentiate between subsets such as T cells, B cells, and natural killer (NK) cells. Therefore, peripheral blood lymphocyte subset enumeration holds significant potential. It can provide a more comprehensive reflection of a patient’s systemic immune status, enabling a more authentic and objective assessment of overall immune activity. This is crucial for precise prognosis identification and guiding treatment strategies (15).

Although peripheral blood lymphocyte subset analysis can simultaneously measure over ten lymphocyte subsets in a patient’s blood, providing rich data for assessing systemic immune status, the processing and interpretation of the resulting multidimensional and complex data remain a significant challenge (16). In this context, machine learning (ML), as a powerful artificial intelligence (AI) method, has been demonstrated to significantly enhance the analysis and interpretation of complex cancer datasets (17, 18). A key advantage of ML methods is their ability to utilize relatively limited data to perform in-depth analysis of the complex biology of diseases and achieve personalized risk stratification, thereby holding promise for improving patient treatment outcomes (19, 20). Despite this clear potential, the application of ML for the deep analysis of OS-specific lymphocyte subset data has not yet been fully explored. This gap extends to the subsequent development of novel biomarkers. Such an endeavor highlights substantial research potential and value in this field.

Based on the aforementioned background, this study aims to construct a prognostic prediction model by integrating peripheral blood lymphocyte subset data with clinicopathological characteristics of OS patients using an ML algorithm. This research seeks to systematically evaluate the independent and synergistic prognostic value of different lymphocyte subsets and explore their combined utility with established clinical indicators. Ultimately, we intend to develop an effective tool capable of accurately identifying high-risk patients and informing individualized treatment strategies, thereby contributing to the advancement of precision medicine practice.

## 2 Patients and methods

### 2.1 Patients

We retrospectively collected the clinical data of OS patients treated at the Musculoskeletal Tumor Center of The First Affiliated Hospital of Zhengzhou University between May 2020 and May 2023. Patient inclusion and exclusion were based on the following criteria: Inclusion criteria were 1) patients with histopathologically confirmed high-grade OS; 2) patients who underwent lymphocyte subset testing before neoadjuvant chemotherapy (NAC); and 3) patients who completed the standard treatment protocol at our hospital. Exclusion criteria were 1) patients with histopathologically confirmed low-grade OS (intramedullary or surface variants) or parosteal osteosarcoma; 2) patients who did not receive the standard treatment (e.g., misdiagnosed, improperly treated, or failed to complete postoperative chemotherapy); 3) patients with hematological diseases; and 4) patients with other malignancies. Ultimately, 65 patients who met all inclusion criteria and did not meet any exclusion criteria were enrolled in the study. All enrolled patients were regularly followed up until death or September 2025, whichever occurred first.

### 2.2 Flow cytometric analysis of peripheral blood lymphocyte subsets

The procedure for peripheral blood lymphocyte subset analysis in this study was conducted as follows. First, prior to the initiation of treatment, blood samples were collected via peripheral venipuncture from patients with pathologically confirmed high-grade OS. Subsequently, the whole blood was treated with a red blood cell (RBC) lysis buffer to remove erythrocytes, resulting in a leukocyte suspension. This leukocyte suspension was then stained strictly following the manufacturer’s protocol using a panel of fluorescently labeled monoclonal antibodies (mAbs) to specifically target distinct lymphocyte surface antigens. The stained samples were analyzed using a BD FACS Canto II flow cytometer for detection and data acquisition. Finally, specialized software was employed to analyze the data, obtaining and recording the absolute counts and/or relative percentages of the following key lymphocyte subsets: CD45+ leukocytes, CD3+ T cells, CD3+CD4+ helper T cells (CD4+ T cells), CD3+CD8+ cytotoxic T cells (CD8+ T cells), CD3-CD56+ NK cells, as well as subsets indicative of T-cell activation and functional status, including CD4+CD28+, CD8+CD28+, CD4+CD38+, CD8+CD38+, CD4+HLA-DR+, and CD8+HLA-DR+ cells.

### 2.3 Development, comparison, and selection of prognostic models

This study integrated traditional statistical methods with various ML algorithms to develop a prognostic prediction model. During the model construction phase, we employed not only stepwise Cox regression models and least absolute shrinkage and selection operator (LASSO) Cox regression as baseline methods but also introduced five ML algorithms for comparative analysis: Random Survival Forest (RSF), Gradient Boosting Machine (GBM), XGBoost, Support Vector Machine (SVM), and K-Nearest Neighbors (KNN). The predictive accuracy of these seven algorithms was systematically evaluated using the concordance index (C-index) and area under the curve (AUC) metrics. Particular emphasis was placed on the clinical utility of the models—prioritizing the model with the fewest variables to balance complexity and practicality, provided predictive performance was maintained. To ensure model reliability, a multiple validation strategy was adopted: Bootstrap validation with 100 resampling iterations was used to assess model stability, 5-fold cross-validation (CV) was employed to evaluate generalizability, and time-dependent receiver operating characteristic (ROC) curve analysis was applied to examine predictive performance at different time points. Ultimately, based on the three dimensions of predictive accuracy, clinical utility, and model reliability, the optimal algorithm for analyzing and interpreting peripheral blood lymphocyte subset data in OS patients was selected. Following model development, the continuous predictive scores were dichotomized into ‘low-risk’ and ‘high-risk’ groups using the median value as the cutoff to facilitate clinical interpretation and application. Partial R code is provided in the **Supplementary Materials**.

### 2.4 Development and validation of the predictive nomogram

A nomogram was constructed based on the independent risk factors identified. First, its discriminatory ability was quantified using the concordance index (C-index). Subsequently, calibration curves were plotted to assess the agreement between predicted probabilities and observed outcomes. Finally, decision curve analysis (DCA) was applied to estimate the clinical net benefit and, consequently, the potential clinical utility of the nomogram.

### 2.5 Comparison of the model’s predictive performance with NLR and PLR

To evaluate and compare prognostic indicators, we calculated NLR and PLR for all patients. Kaplan-Meier survival analysis was used to compare overall survival between high- and low-risk groups (stratified by the median value) for each inflammatory index. This was followed by univariate Cox regression analysis to assess the association of NLR and PLR (as continuous variables and as risk groups) with overall survival in osteosarcoma. Finally, ROC analysis was performed to compare the predictive capabilities of these traditional markers with our model-derived score.

### 2.6 Statistical analysis

All statistical analyses and visualizations in this study were performed using R software (version 4.4.2). The normality of continuous variables was assessed using the Shapiro-Wilk test. Data conforming to a normal distribution are presented as mean ± standard deviation (mean ± SD), while non-normally distributed (skewed) data are expressed as median (range). For group comparisons, appropriate statistical tests were selected based on the distribution characteristics of the data: the independent samples t-test was used for normally distributed data, and the Mann-Whitney U test was employed for non-normally distributed data. Categorical data are presented as actual counts (frequencies), and comparisons between groups were performed using the Chi-square test or Fisher’s exact test, as appropriate. Overall survival (OS) curves were generated using the Kaplan-Meier (K-M) method and compared using the log-rank test. A two-tailed test was adopted for all analyses, and a P-value < 0.05 was considered statistically significant.

## 3 Results

### 3.1 Patient characteristics

A total of 65 patients with OS were included in this study, comprising 30 males and 35 females, with a median age of 16 years (range: 7–65 years). Regarding tumor characteristics, the primary tumor was located in the lower limbs in 59 patients and in the upper limbs in 6 patients. At initial diagnosis, 43 patients had a tumor size < 8 cm, while 22 patients had a tumor size ≥ 8 cm. Metastasis was present at diagnosis in 13 patients, whereas 52 patients showed no evidence of metastasis. Pathological fracture was identified at initial presentation in 6 patients. Based on body mass index (BMI) assessment, 37 patients were classified as having a normal BMI, and 28 were classified as abnormal. By the last follow-up, 17 patient deaths had been recorded (**Table 1**).

**Table 1:** Demographic and clinical characteristics of osteosarcoma patients (n=65).

Characteristic	Category	Patients (n, %)
Gender	Male	30 (46.2%)
	Female	35 (53.8%)
Median Age (Range)		16 years (7–65)
Location	Lower Limbs	59 (90.8%)
	Upper Limbs	6 (9.2%)
Tumor size	< 8 cm	43 (66.2%)
	≥ 8 cm	22 (33.8%)
Primary metastases	Yes	13 (20.0%)
	No	52 (80.0%)
Pathological fracture	Yes	6 (9.2%)
	No	59 (90.8%)
BMI	Normal	37 (56.9%)
	Abnormal	28 (43.1%)

### 3.2 Characterization of peripheral blood lymphocyte subsets

**Figure 1:**
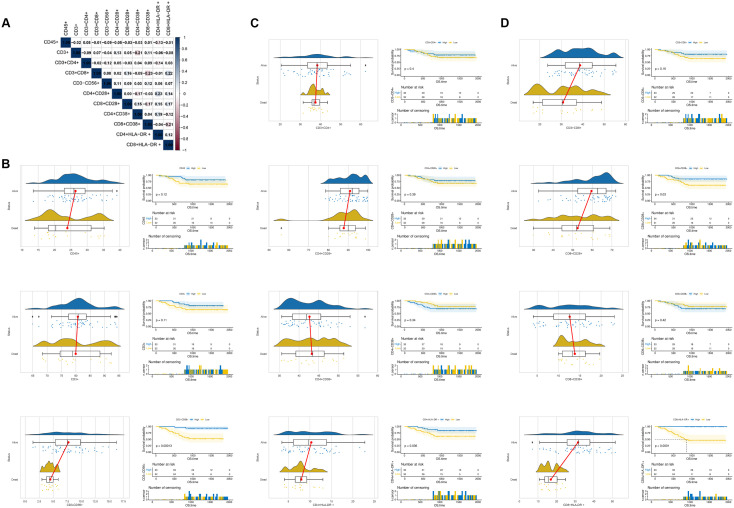
Comprehensive analysis of peripheral blood lymphocyte subsets and their prognostic value in osteosarcoma patients. **(A)** Correlation matrix of the 11 lymphocyte subsets based on surface marker expression profiles; **(B)** Comparison of CD45+ total lymphocytes, CD3+ T cells, and CD3−CD56+ NK cells between deceased and survival groups using raincloud plots (left), which combine half-violin plots (density distribution), boxplots, and scatterplots; K-M survival curves (right) stratify patients by median expression of each subset. **(C)** Raincloud plots and survival curves for CD4+ T cells and their functional subsets (CD4+CD28+, CD4+CD38+, CD4+HLA-DR+). **(D)** Raincloud plots and survival curves for CD8+ T cells and their functional subsets (CD8+CD28+, CD8+CD38+, CD8+HLA-DR+).

Through flow cytometric analysis, this study classified lymphocytes into 11 subsets based on their surface marker expression profiles, comprising the total lymphocyte percentage, T-cell percentage, NK-cell percentage, and eight functional T-cell subset percentages. **Figure 1A** illustrates the correlations among the 11 lymphocyte subsets. Comparative analysis revealed that the proportions of CD4^+^CD38^+^ and CD8^+^CD38^+^ subsets were higher in the deceased group than in the survival group, whereas the majority of the remaining lymphocyte subsets were lower in the deceased group (**Figures 1B-D**). It is noteworthy that, among the subsets showing numerical differences between the two groups, the differences in five subsets—CD45^+^, CD3^+^, CD3^+^CD4^+^, CD4^+^CD38^+^, and CD8^+^CD38^+^—did not reach statistical significance. Patients were stratified into high-expression and low-expression groups based on the median value of each lymphocyte subset, and K-M survival curves were plotted for comparison. The analysis revealed that patients with higher proportions of CD3^-^CD56^+^ NK cells, CD4^+^HLA-DR^+^ activated helper T cells, CD8^+^CD28^+^ proliferative cytotoxic T cells, and CD8^+^HLA-DR^+^ activated cytotoxic T cells in peripheral blood exhibited significantly prolonged overall survival (**Figures 1B–D**, P<0.05).

### 3.3 Feature selection and performance of the GBM prognostic model

**Figure 2:**
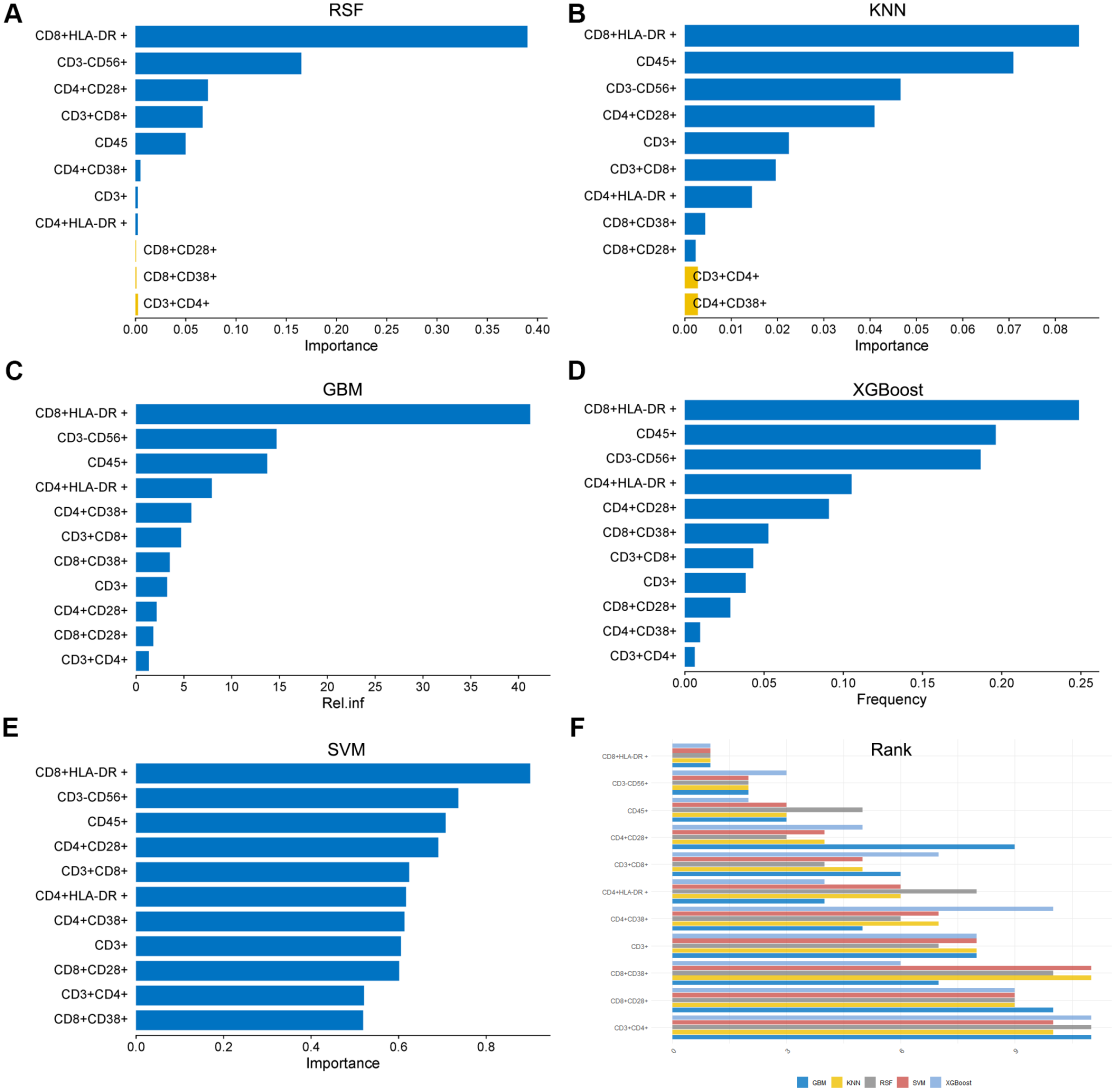
Variable importance of peripheral blood lymphocyte subsets evaluated by five ML algorithms for prognostic prediction in osteosarcoma. **(A)** RSF; **(B)**KNN; **(C)** GBM; metric: relative influence [Rel.inf]; **(D)** XGBoost (metric: Frequency); **(E)** SVM. In bar plots **(A–E)**, blue bars indicate positive importance values, while yellow bars (where applicable) represent negative contributions. All subsets in panels (Cnel showed exclusively positive values. **(F)** Consolidated ranking of the 11 lymphocyte subsets across all five algorithms.

This study initially employed stepwise regression and LASSO regression as baseline models, which were compared against five ML algorithms: RSF, XGBoost, GBM, SVM, and KNN. By systematically applying five machine learning algorithms to evaluate the variable importance of 11 peripheral blood lymphocyte subsets, this study elucidated the prognostic value of key subsets. Notably, CD8^+^HLA-DR^+^ activated cytotoxic T cells consistently ranked first in variable importance across all five algorithms, underscoring their robustness as a core prognostic biomarker. The CD3^-^CD56^+^ NK cell subset ranked second in four algorithms (GBM, KNN, SVM, and RSF) but third in the XGBoost algorithm. Furthermore, the CD45^+^ total leukocyte subset ranked second in the XGBoost algorithm, third in the GBM, KNN, and SVM algorithms, and dropped to fourth in the RSF algorithm, reflecting fluctuations in its importance across different models (**Figures 2A–F**). These findings collectively highlight the critical role of multi-algorithm consensus in screening robust feature subsets to enhance the reliability of prognostic models.

**Figure 3:**
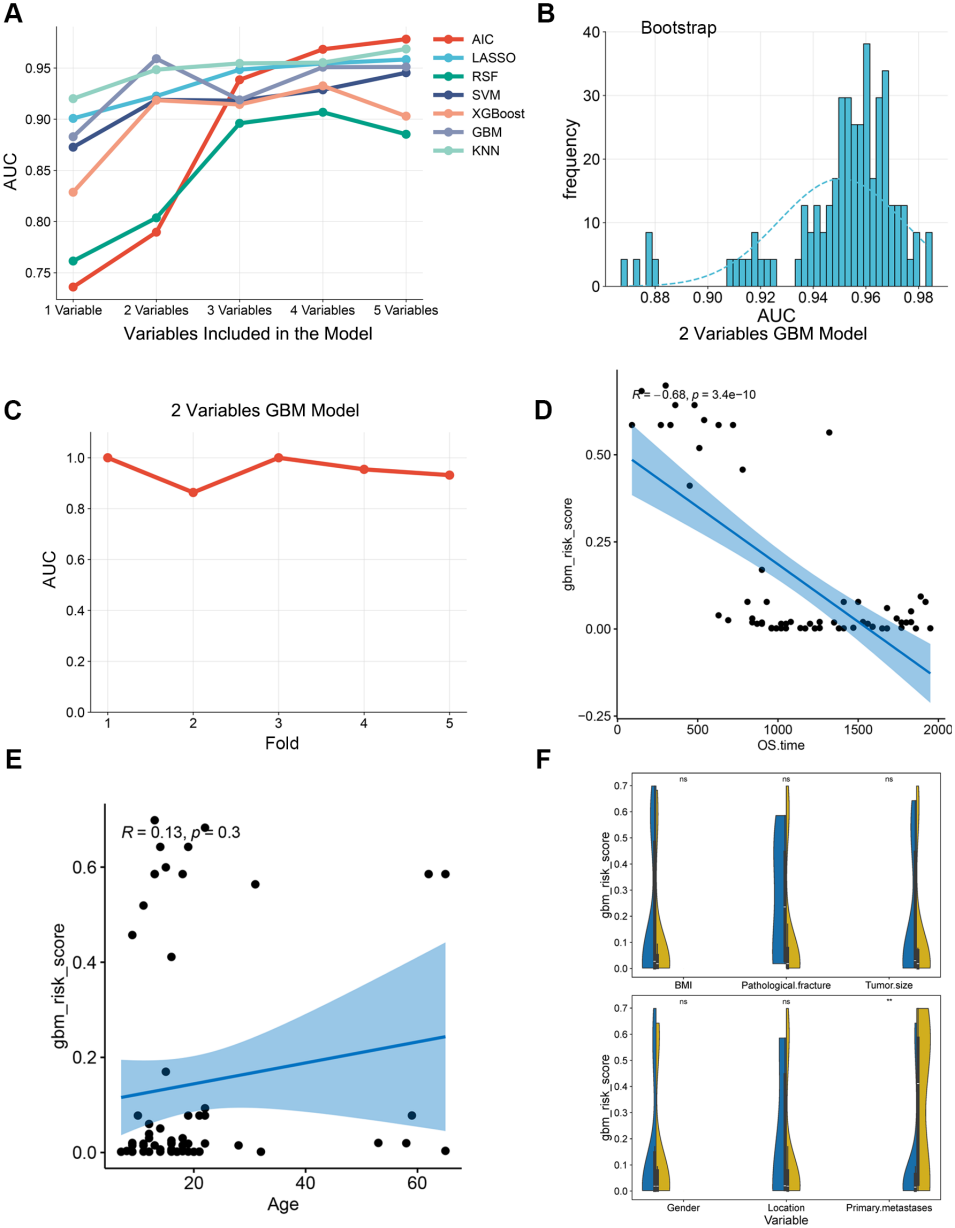
Model selection process and comprehensive evaluation of the final GBM prognostic model for osteosarcoma. **(A)** Line graph comparing the AUC values of models constructed by LASSO, SVM, XGBoost, GBM, and KNN algorithms across variable combinations (1 to 5 features). **(B)** Histogram with fitted curve showing the frequency distribution of AUC values from 100 bootstrap validation iterations **(C)** AUC value derived from 5-fold cross-validation. **(D)** Scatter plot demonstrating a significant negative correlation between the gbm_risk_score and overall patient survival time. **(E)** Scatter plot showing no significant correlation between gbm_risk_score and patient age. **(F)** Violin plots comparing gbm_risk_score across subgroups defined by key clinical variables (e.g., age, gender, tumor location).

Through systematic evaluation of predictive performance from single-variable to five-variable combination models, it was found that the two-variable models constructed by LASSO, SVM, XGBoost, GBM, and KNN all achieved AUC values above 0.90. Among these, the GBM two-variable model demonstrated the best performance (AUC = 0.9591), whereas the two-variable models for RSF and stepwise regression yielded AUC values of 0.837 and 0.7897, respectively (**Figure 3A**). Further analysis indicated that, except for Akaike information criterion (AIC) and RSF, increasing the number of variables provided limited improvement in AUC for the other algorithms. Consequently, balancing predictive power and model simplicity, the GBM two-variable model was ultimately selected for analyzing OS lymphocyte subset data. The features incorporated into the model were the CD3^-^CD56^+^ cell subset and the CD8^+^HLA-DR^+^ cell subset, with the model parameters set as n.trees = 50, interaction.depth = 3, and shrinkage = 0.1. Both Bootstrap validation and 5-fold CV confirmed the model’s good stability (**Figures 3B, C**). Furthermore, the gbm_risk_score derived from the model showed a significant negative correlation relationship with overall patient survival time (**Figure 3D**, R = -0.68, P < 0.001). To evaluate the clinical relevance of the gbm_risk_score, this study further investigated its relationship with key clinical variables. The analysis revealed that patients with metastases at initial diagnosis had a significantly higher gbm_risk_score compared to those without metastases (P = 0.001). However, no statistically significant differences in the score were observed across subgroups defined by other clinical variables, such as age, gender, or tumor location (**Figures 3E, F**).

### 3.4 The GBM risk score as a powerful and independent prognostic indicator

**Figure 4:**
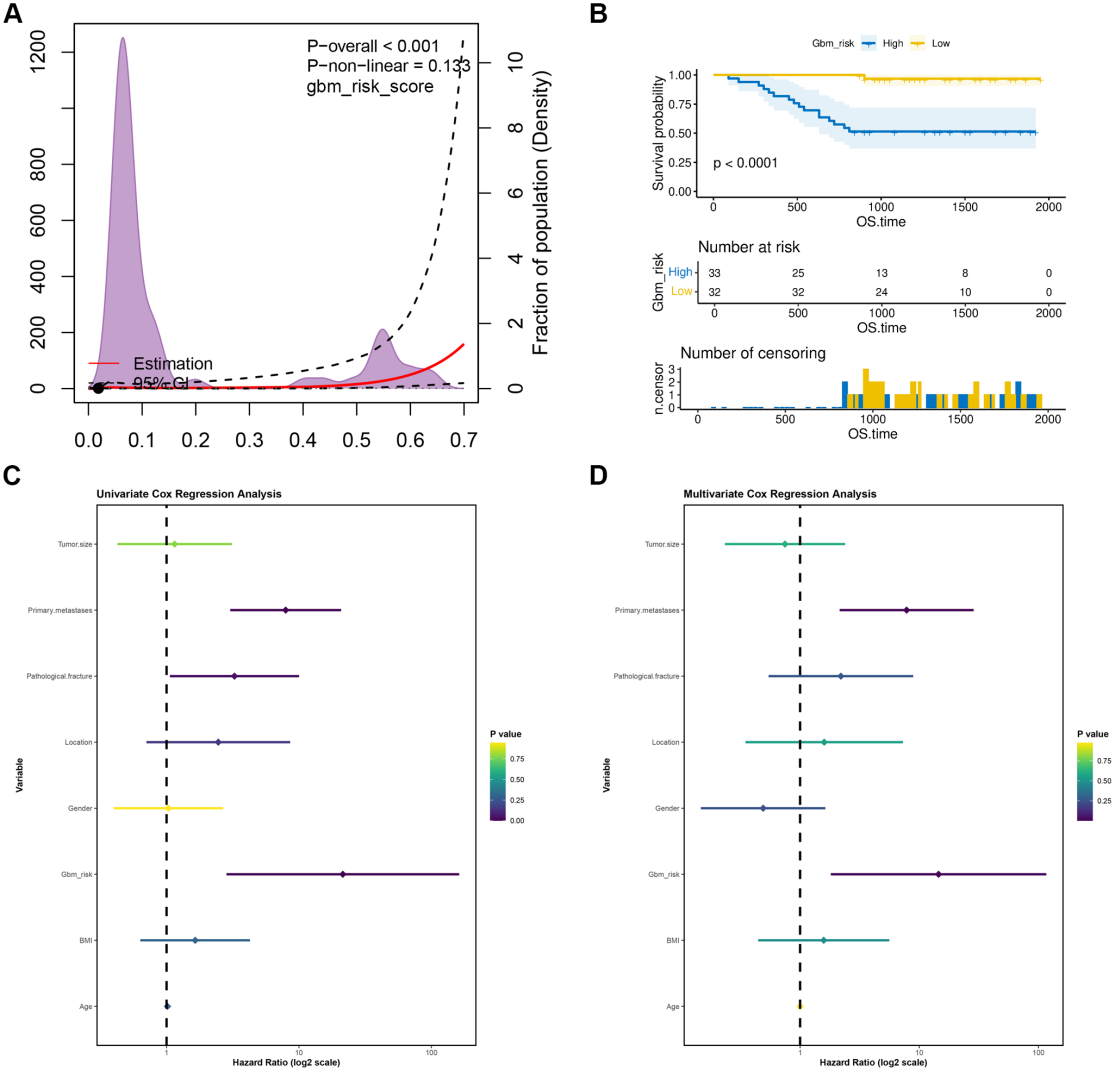
Prognostic value of the GBM risk group in predicting overall survival of osteosarcoma patients. **(A)** Restricted cubic spline analysis of the gbm_risk_score for overall survival. **(B)** K-M curves of low- and high-risk groups stratified by the median gbm_risk_score. **(C)** Forest plot of univariate Cox analysis for osteosarcoma overall survival. **(D)** Forest plot of multivariate Cox analysis for osteosarcoma overall survival.

Analysis using restricted cubic spline interpolation demonstrated a linear relationship between the gbm_risk_score and the risk of overall survival in osteosarcoma patients (P = 0.133). Subsequently, patients were stratified into low-risk and high-risk groups based on the median value of this score. K-M survival analysis results clearly indicated that the overall survival time was significantly shorter in the high-risk group compared to the low-risk group (P < 0.001), validating the definite discriminatory ability of the gbm_risk_score in clinical prognostic stratification (**Figures 4A, B**). To explore the potential clinical application value of the GBM risk score, this study conducted a comprehensive analysis integrating it with various clinical characteristics. Univariate Cox regression analysis revealed that the GBM risk grouping (hazard ratio [HR] = 21.437, P = 0.003), metastasis status at initial diagnosis (HR = 7.934, P < 0.001), and pathological fracture (HR = 3.257, P = 0.039) were significantly associated with the OS of OS patients. Subsequently, multivariate Cox regression analysis further confirmed that the GBM risk grouping (HR = 14.516, P = 0.012) and metastasis status at initial diagnosis (HR = 7.831, P = 0.002) were independent risk factors affecting patient prognosis (**Figures 4C, D**). These findings indicate that the GBM risk score, based on lymphocyte subsets, holds prognostic predictive value independent of traditional clinical indicators.

### 3.5 Development and clinical validation of a prognostic nomogram

**Figure 5:**
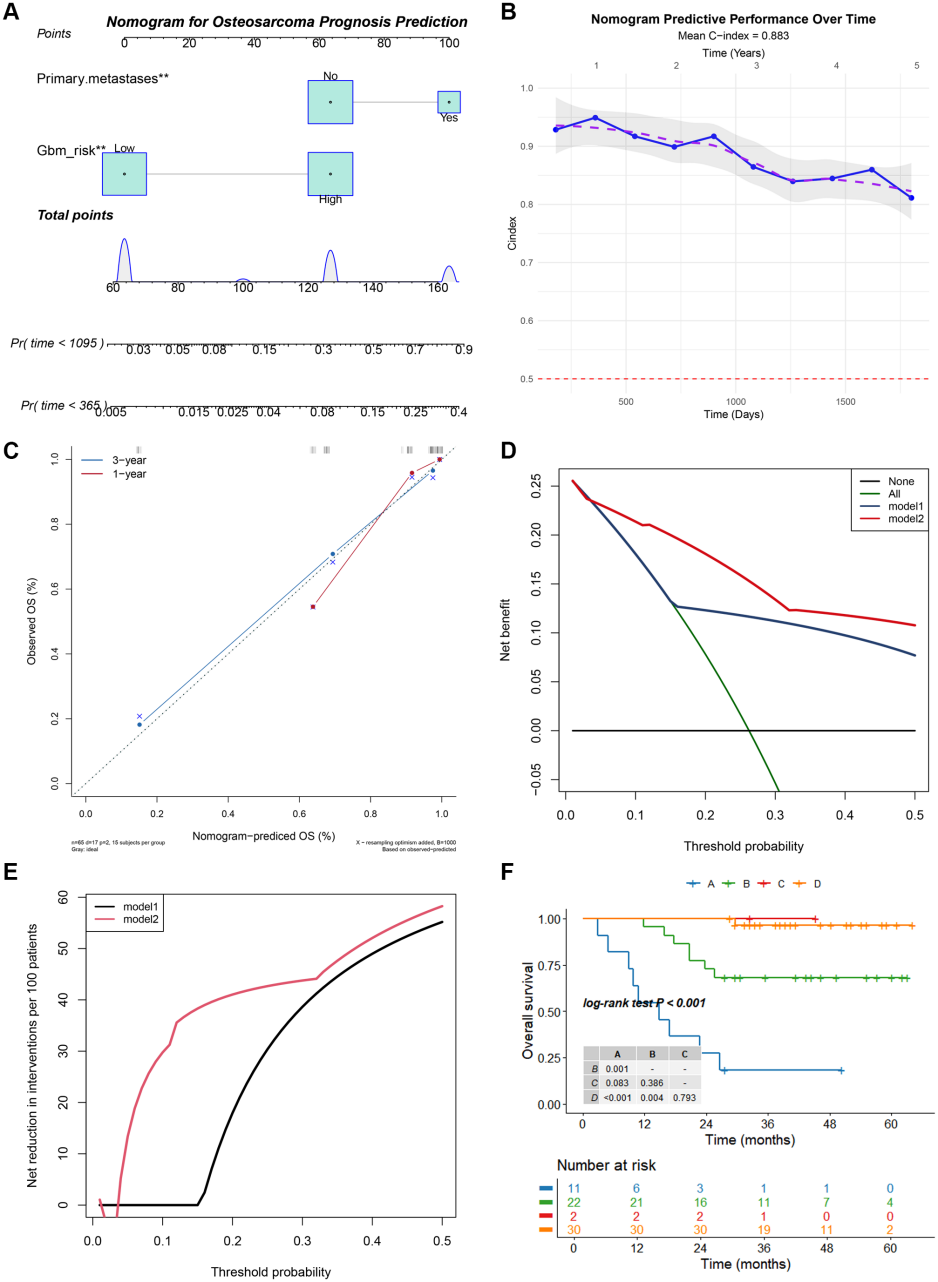
Integration of the GBM risk group with clinical variables for prognostic prediction in osteosarcoma. **(A)** Prognostic nomogram developed by combining the GBM risk grouping and primary metastasis status. **(B)** C-index of the nomogram. **(C)** Calibration curves for 1- and 3-year OS. **(D, E)** DCA showing the clinical net benefit and net reduction **(F)** K-M survival curves of patient subgroups stratified by the GBM risk group and primary metastases.

Subsequently, a prognostic nomogram prediction model was developed based on the GBM risk grouping and primary metastasis status (**Figure 5A**). This model demonstrated excellent predictive performance, achieving a C-index of 0.883, indicating high discriminative accuracy (**Figure 5B**). Furthermore, the calibration curve confirmed that the nomogram’s predicted 3-year OS probabilities showed the best agreement with the actual observed outcomes (**Figure 5C**). DCA revealed that the incorporation of the GBM risk grouping provided significant clinical net benefit across a wide range of threshold probabilities (**Figures 5D, E**). Additionally, by combining the GBM risk grouping with primary metastasis status in K-M survival curves, a patient subgroup with a higher risk of mortality was successfully identified among those without metastases at initial diagnosis, thereby highlighting the model’s refined risk-stratification capability (**Figure 5F**).

### 3.6 The GBM risk score demonstrates superior predictive performance to NLR and PLR

**Figure 6:**
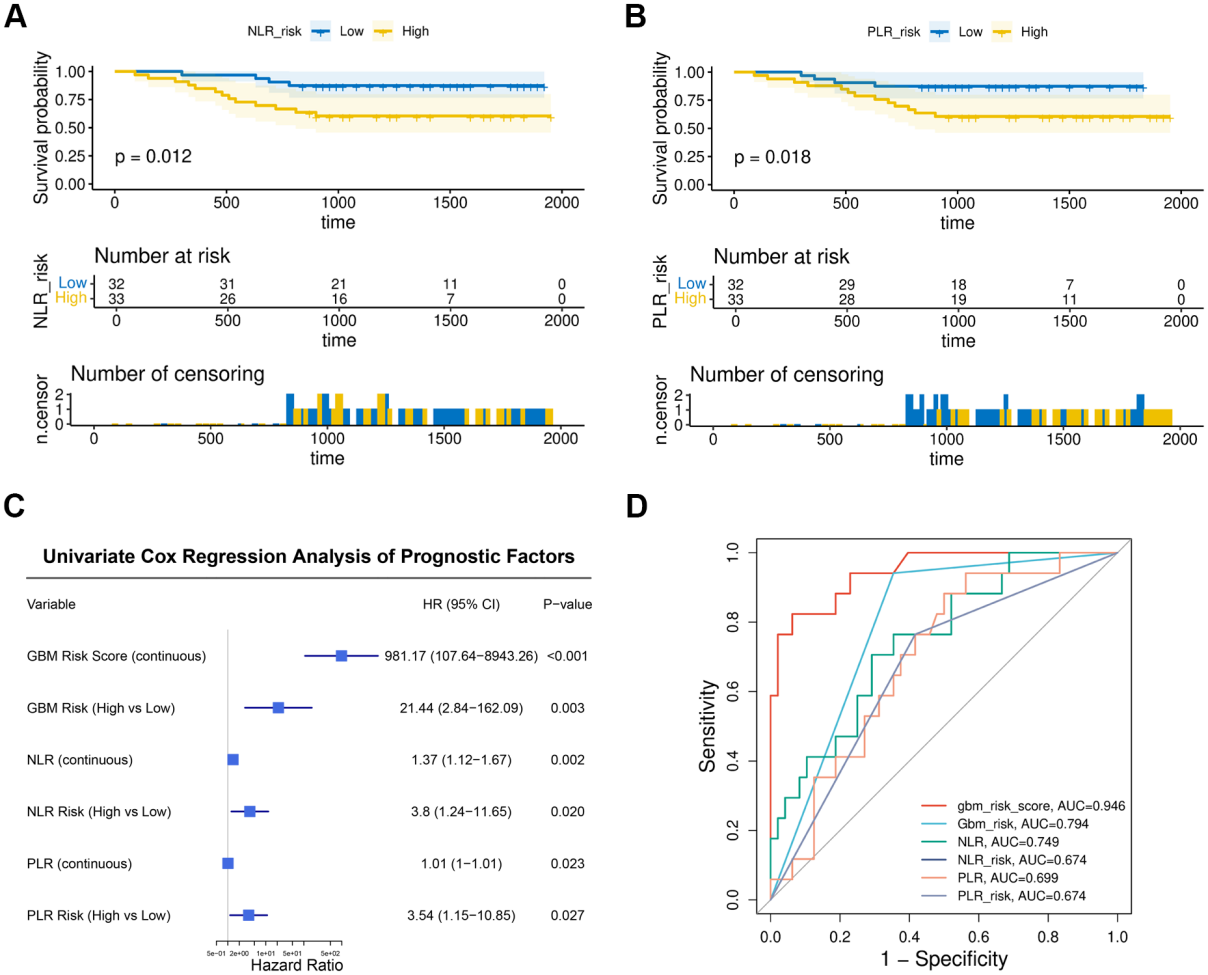
The GBM prognostic model exhibited superior predictive capability compared to NLR and PLR. **(A)** K-M curves of low- and high-risk groups stratified by the median NLR. **(B)** K-M curves of low- and high-risk groups stratified by the median PLR. **(C)** Forest plot of univariate Cox analysis for osteosarcoma overall survival. **(D)** The ROC curves illustrate the performance of GBM, NLR, and PLR in predicting the 3-year overall survival of patients.

As shown in **Figures 6A, B**, Kaplan-Meier analysis confirmed that patients in the high-risk groups for both NLR and PLR exhibited shorter OS than those in the corresponding low-risk groups (NLR: P = 0.012; PLR: P = 0.018). Univariate Cox analysis indicated that NLR (HR = 1.37, P = 0.002), PLR (HR = 1.01, P = 0.023), and their dichotomized risk categories were all significantly associated with OS in patients with osteosarcoma (**Figure 6C**). ROC analysis revealed that both the continuous gbm_risk_score and its dichotomized risk classification had significantly better predictive performance for OS than either the NLR or the PLR in predicting 3-year OS (**Figure 6D**). These results suggest that the lymphocyte subset-based GBM risk score may offer superior prognostic value compared to traditional peripheral blood inflammatory indices.

## 4 Discussion

Through a systematic analysis of peripheral blood lymphocyte subsets in 65 OS patients, this study revealed significant associations between immune characteristics of lymphocytes and patient prognosis. It was found that the distribution of lymphocyte subsets differed significantly between deceased and surviving patient groups. Specifically, the proportions of six subsets—CD3^+^CD8^+^, CD3^-^CD56^+^, CD4^+^CD28^+^, CD8^+^CD28^+^, CD4^+^HLA-DR^+^, and CD8^+^HLA-DR^+^—were significantly lower in the deceased group compared to the survival group. Notably, although an increasing trend was observed in the proportions of the CD4^+^CD38^+^ and CD8^+^CD38^+^ subsets within the deceased group, this difference did not reach statistical significance. By comparing various ML algorithms, a prognostic prediction model based on the GBM was established. This model, utilizing only two variables—CD3^-^CD56^+^ NK cells and CD8^+^HLA-DR^+^ activated cytotoxic T cells—achieved excellent predictive performance, with an AUC of 0.9591. Furthermore, the gbm_risk_score, constructed based on these two lymphocyte subsets, showed a significant negative correlation with overall patient survival and was confirmed as an independent prognostic factor in multivariate analysis, distinct from traditional clinical factors such as metastasis status at initial diagnosis. The subsequently developed nomogram model demonstrated good accuracy and clinical utility in predicting the 3-year OS rate, successfully identifying high-risk subgroups among patients without initial metastasis. These findings provide new perspectives and methodologies for the immune prognostic assessment of OS.

Previous studies have extensively explored the role of total peripheral blood lymphocyte count and its derived biomarkers, such as the NLR, in prognostic assessment across various cancers (21–23). For instance, in extensive-stage small cell lung cancer (ES-SCLC), research has demonstrated that patients with a low pretreatment total lymphocyte count or a high NLR exhibit shorter OS (24). Similarly, a meta-analysis focusing on gastric cancer (GC) patients revealed that elevated NLR and platelet-to-lymphocyte ratio (PLR) were associated with poorer OS and progression-free survival (PFS) following treatment with immune checkpoint inhibitors (ICIs), whereas a high lymphocyte-to-monocyte ratio (LMR) was correlated with improved survival outcomes (25). Furthermore, in OS research, NLR has been confirmed as an independent risk factor for predicting patient survival, and dynamic monitoring of NLR changes can further enhance the accuracy of prognostic evaluation (10). Collectively, these findings underscore the significant value of total peripheral blood lymphocyte count and its derived ratios in pan-cancer prognostic prediction and even in the evaluation of treatment response.

With the deepening of research on the tumor immune microenvironment (TIME), the functional diversity of lymphocyte subsets and their complex roles in tumorigenesis and development have become increasingly clear (26). Among them, CD4+ T cells are crucial for initiating and regulating adaptive immunity; they effectively promote anti-tumor immune responses by supporting the clonal expansion, differentiation, and memory formation of cytotoxic T lymphocytes (CTLs) (27, 28). CD8+ T cells, as direct effector cells, kill tumor cells through the release of cytotoxic substances such as perforin and granzymes (29). In contrast, Tregs play an immunosuppressive role in the TIME by inhibiting the function of effector T cells, thereby promoting tumor immune escape and progression (30); studies have shown that the proportion of Tregs in the peripheral blood of some cancer patients is significantly higher than that in healthy individuals (31). Therefore, compared to measuring the total lymphocyte count alone, precise analysis of these functionally diverse and often antagonistic lymphocyte subsets in peripheral blood provides a more comprehensive reflection of the overall immune status of cancer patients and demonstrates greater potential for clinical application in prognostic prediction.

CD56+ NK cells, one of the cornerstones of the gbm_risk_score, constitute a central component of innate anti-tumor immunity, which can directly lyse tumor cells without the need for prior activation, notably those with low or absent MHC-I expression (32). Accumulating evidence indicates that the presence of NK cells is generally associated with a favorable prognosis. For instance, in patients with advanced hepatocellular carcinoma treated with ICIs, an increased proportion of peripheral blood NK cells at week 3 post-treatment is an independent predictor of objective response and long-term survival (PFS and OS) (33). Similarly, in advanced gastric cancer patients treated with apatinib, a peripheral blood NK cell proportion below 17% is associated with poorer PFS and OS (34). In osteosarcoma, a dual-center retrospective study of 106 patients demonstrated that a high proportion of NK cells is correlated with improved survival (35). Furthermore, preclinical strategies aimed at enhancing NK cell migration and infiltration into osteosarcoma tumors have yielded promising preliminary results (36). These findings align with our own data, thereby highlighting the critical role of NK cells in tumor immune surveillance via direct cytotoxicity.

CD8^+^HLA-DR^+^ T cells represent a subset of CTLs commonly regarded as markers of activated, effector memory T cells (37). HLA-DR is a class II MHC molecule whose expression on T cells typically denotes an activated state associated with antigen presentation or effector functions. Existing research suggests that CD8^+^HLA-DR^+^ T cells can have dual prognostic significance in cancer patients. For example, studies in breast cancer show that high levels of HLA-DR^+^ CTLs are associated with a favorable response to neoadjuvant chemotherapy and improved PFS, indicating a positive prognostic role (38, 39). However, other research indicates that in primary central nervous system lymphoma, elevated levels of CD8^+^HLA-DR^+^ T cells has been linked to T cell exhaustion and an immunosuppressive microenvironment, suggesting a poor prognosis (37). In contrast, the prognostic value of CD8^+^HLA-DR^+^ T cells in osteosarcoma has not been definitively established. Only one study has proposed that increased HLA-DR expression in osteosarcoma likely reflects an ongoing anti-tumor immune response (40). In our study, a higher proportion of peripheral blood CD8^+^HLA-DR^+^ T cells was associated with prolonged OS. This implies that within the context of osteosarcoma, this cell population may represent an effective anti-tumor immune response rather than an exhausted phenotype. Further studies are warranted to validate this finding.

While numerical differences were observed for several lymphocyte subsets (including CD45^+^, CD3^+^, CD3^+^CD4^+^, CD4^+^CD38^+^, and CD8^+^CD38^+^) between deceased and surviving patients, these differences did not attain statistical significance in our cohort. Kaplan-Meier analysis also revealed no significant difference in OS between the defined risk groups. Several factors may account for these non-significant findings. First, the relatively limited sample size may have reduced the statistical power to detect associations with modest clinical effect sizes. Furthermore, the use of the median value as a uniform cutoff for all biomarkers—a conservative strategy adopted to mitigate overfitting in a small cohort—may have obscured the predictive utility of some markers. Consequently, validation of these results will require future multi-center studies with larger sample sizes or well-designed prospective trials.

In contrast to the non-significant markers, our analysis identified several lymphocyte subsets with clear prognostic value. Patients with higher proportions of CD8^+^CD28^+^ T cells had significantly longer OS, a finding consistent with reports by Liu et al. in non-small cell lung cancer (NSCLC) that affirm the central role of these effector T cells in anti-tumor immunity (41, 42). Furthermore, activated CD4^+^HLA-DR^+^ T cells were significantly enriched in the survival group and independently predicted a better prognosis. The importance of such highly activated T cells has also been validated in studies on NSCLC and in melanoma cohorts receiving anti-PD-1 therapy, further emphasizing the key value of activated T cells in anti-tumor immunity (43, 44). Collectively, these results underscore the prognostic significance of specific activated T-cell populations.

The biological interpretation of complex omics data has long been a primary challenge in clinical translational research (16). The emergence of ML algorithms provides an effective tool for discovering potential biomarkers from these high-dimensional datasets and constructing predictive models (45–47). For instance, a 10-metabolite GC diagnostic model developed by Chen et al. using ML analysis demonstrated performance significantly superior to models based on traditional clinical parameters (16). In OS research, Zhao et al. successfully developed a diagnostic model for identifying high-risk subtypes by integrating transcriptomic and methylation data with ML algorithms, which showed good diagnostic performance (48). To optimize the model and avoid overfitting, this study initially employed seven different algorithms to construct models containing combinations of 1 to 5 variables. The results revealed that algorithms such as GBM and XGBoost achieved AUC values exceeding 0.90 with only two variables, substantially outperforming the traditional AIC algorithm. This highlights the advantage of ML in data dimensionality reduction and building streamlined yet robust prognostic models. The stability of the model was further confirmed through internal Bootstrap validation and 5-fold CV. Ultimately, the risk grouping defined by integrating lymphocyte subsets via ML was confirmed as an independent prognostic factor for OS patients. Its significant clinical value lies in its ability to identify a subgroup of patients without detectable metastasis at initial diagnosis who nonetheless harbor a high risk of mortality.

It is important to acknowledge the limitations of this study. First, the retrospective and single-center nature of the cohort (n=65 high-grade osteosarcoma patients) introduces inherent risks of selection bias and limits the external generalizability of the findings. While osteosarcoma is a rare disease, making large cohorts challenging to assemble, and the institution’s peripheral blood lymphocyte subset analysis was only recently implemented, these factors underscore the need for cautious interpretation. Furthermore, despite employing machine learning algorithms (GBM) and rigorous internal validation strategies (bootstrap and 5-fold cross-validation) to enhance model robustness and mitigate overfitting risks in this modest dataset, the absence of an independent external validation cohort remains a critical constraint. Future validation through larger, multicenter prospective studies is indispensable to confirm the generalizability and clinical utility of the prognostic model. Second, regarding the lymphocyte subset analysis, the panel used in our institution’s flow cytometry did not include Tregs, a crucial subset, preventing the assessment of their role in OS prognosis. Furthermore, with the growing understanding of the TIME, characterizing T-cell function using only dual markers such as CD4+CD38+ or CD8+CD38+ may be insufficient. For instance, studies indicate that CD8+CD38+ programmed cell death protein 1-positive (PD-1+) and PD-1− subpopulations may possess distinct functional properties (49).

Nevertheless, by integrating lymphocyte subset data with ML algorithms, this study effectively revealed the potential value of peripheral blood lymphocyte subsets in prognostic assessment for OS. The findings suggest that functional lymphocyte subsets (e.g., activated or exhausted phenotypes) might reflect a patient’s immune status and disease progression risk more accurately than the simple CD4/CD8 ratio alone. Future research should integrate a broader panel of markers (e.g., PD-1, cytotoxic T-lymphocyte-associated protein 4 [CTLA-4]) to deeply characterize lymphocyte functional subsets and clarify their specific roles in personalized prognosis prediction and treatment strategies for OS.

## 5 Conclusion

In conclusion, this research introduces a pioneering framework for OS prognosis by leveraging ML to decode peripheral blood lymphocyte subset signatures. This model addresses the shortcomings of conventional prognostic markers and provides a robust theoretical and practical basis for future investigations into the evolving TIME, ultimately aiming to improve clinical decision-making and patient survival.

## Data Availability

The raw data supporting the conclusions of this article will be made available by the authors, without undue reservation.
