# Supplementary material for: A machine learning-driven prognostic model based on peripheral blood lymphocyte subsets in osteosarcoma
Source: Front Immunol. 2026 Jan 28;17:1733518. doi: 10.3389/fimmu.2026.1733518 (PMC12891181; doi:10.3389/fimmu.2026.1733518)
Supplement: Supplementary file 1 [file DataSheet1.docx]

library(survival)

library(survminer)

library(gbm) # GBM

library(xgboost) # XGBoost

library(caret)

library(timeROC)

library(ggplot2)

library(pROC)

library(dplyr)

# Create Results Directory

results_dir <- "GBM_XGBoost_Model"

if (!dir.exists(results_dir)) {

dir.create(results_dir)

}

# 1. Data Preparation and Preprocessing

cat("=== Data Preparation and Preprocessing ===\n")

# Check data

cat("Data dimensions:", dim(rt), "\n")

cat("Survival status distribution:\n")

table(rt$OS)

# Convert survival time to years

rt$OS.time_years <- rt$OS.time / 365.25

# Create binary variable for survival outcome

# Use median survival time as cutoff

median_survival <- median(rt$OS.time[rt$OS == 1], na.rm = TRUE)

rt$survival_class <- ifelse(rt$OS.time > median_survival, "长期生存", "短期生存")

rt$survival_class[rt$OS == 0] <- "长期生存" # Censored data considered as long-term survival

cat("Median survival time:", median_survival, "days\n")

cat("Distribution of survival classes:\n")

table(rt$survival_class)

# Prepare predictor variables

predictor_names <- paste0("Variable", 1:11)

cat("Initial number of predictor variables:", length(predictor_names), "\n")

# 2. GBM Model - Pursuing Optimal Cost-Effectiveness

cat("\n=== GBM Model - Pursuing Optimal Cost-Effectiveness ===\n")

# Prepare GBM input data

gbm_data <- rt[, c(predictor_names, "survival_class")]

# Convert response variable to numeric (0/1)

gbm_data$survival_class_numeric <- ifelse(gbm_data$survival_class == "短期生存", 1, 0)

# Train initial GBM model to obtain feature importance

cat("Training initial GBM model to obtain feature importance...\n")

gbm_base <- gbm(

survival_class_numeric ~ .,

data = gbm_data[, c(predictor_names, "survival_class_numeric")],

distribution = "bernoulli",

n.trees = 100,

interaction.depth = 3,

shrinkage = 0.1,

cv.folds = 3,

verbose = FALSE

)

# Get feature importance

gbm_imp <- summary(gbm_base, plotit = FALSE)

cat("GBM feature importance ranking:\n")

print(gbm_imp)

# Save feature importance

write.csv(gbm_imp, file.path(results_dir, "GBM_initial_feature_importance.csv"), row.names = FALSE)

# 3. Identifying Optimal GBM Variable Combination

cat("\n=== Identifying Optimal GBM Variable Combination ===\n")

# Set up cross-validation

ctrl <- trainControl(

method = "cv",

number = 5,

classProbs = TRUE,

summaryFunction = twoClassSummary,

savePredictions = "final"

)

# Set up parameter grid (simplified version)

gbmGrid <- expand.grid(

n.trees = c(50, 100), # Number of iterations

interaction.depth = c(3, 6), # Interaction depth

shrinkage = c(0.01, 0.1), # Learning rate

n.minobsinnode = 10 # Minimum number of observations in terminal nodes

)

# Test different numbers of important variables

gbm_performance <- data.frame()

best_gbm_models <- list()

max_vars <- min(6, nrow(gbm_imp)) # Test up to 6 variables

for (n_vars in 1:max_vars) {

# Select the top n_vars most important variables

current_vars <- gbm_imp$var[1:n_vars]

cat("Testing", n_vars, "variables:", paste(current_vars, collapse = ", "), "\n")

# Prepare data subset

current_data <- gbm_data[, c(current_vars, "survival_class")]

current_data$survival_class <- as.factor(current_data$survival_class)

# Train GBM model

gbm_model <- train(

survival_class ~ .,

data = current_data,

method = "gbm",

trControl = ctrl,

tuneGrid = gbmGrid,

metric = "ROC",

verbose = FALSE

)

# Get best parameters

best_tune <- gbm_model$bestTune

cat("Best parameters: n.trees=", best_tune$n.trees,

"interaction.depth=", best_tune$interaction.depth,

"shrinkage=", best_tune$shrinkage, "\n")

# Predict on training set

gbm_pred <- predict(gbm_model, newdata = current_data, type = "prob")

current_auc <- gbm_model$results %>%

filter(n.trees == best_tune$n.trees,

interaction.depth == best_tune$interaction.depth,

shrinkage == best_tune$shrinkage) %>%

pull(ROC)

cat("Cross-validated AUC:", round(current_auc, 4), "\n")

# Record results

gbm_performance <- rbind(gbm_performance,

data.frame(

VariableCount = n_vars,

AUC = current_auc,

Variables = paste(current_vars, collapse = ", "),

n.trees = best_tune$n.trees,

interaction.depth = best_tune$interaction.depth,

shrinkage = best_tune$shrinkage

))

# Save best model

best_gbm_models[[as.character(n_vars)]] <- gbm_model

}

# 4. Select Optimal GBM Cost-Effectiveness Model

cat("\n=== Select Optimal GBM Cost-Effectiveness Model ===\n")

# Calculate cost-effectiveness metric (AUC / number of variables)

gbm_performance$Efficiency <- gbm_performance$AUC / gbm_performance$VariableCount

# Set performance threshold (acceptable AUC drop)

performance_threshold <- 0.03

max_auc <- max(gbm_performance$AUC)

# Find the minimum variable set meeting performance requirements

viable_gbm <- gbm_performance %>%

filter(AUC >= max_auc - performance_threshold) %>%

arrange(VariableCount)

if (nrow(viable_gbm) > 0) {

# Select the model with the fewest variables

optimal_gbm <- viable_gbm[1, ]

} else {

# If none meet the condition, select the model with the best cost-effectiveness

optimal_gbm <- gbm_performance[which.max(gbm_performance$Efficiency), ]

}

cat("\n★ Optimal GBM Cost-Effectiveness Model Configuration:\n")

cat("Number of variables:", optimal_gbm$VariableCount, "\n")

cat("Variables:", optimal_gbm$Variables, "\n")

cat("AUC:", round(optimal_gbm$AUC, 4), "\n")

cat("Cost-effectiveness metric:", round(optimal_gbm$Efficiency, 4), "\n")

# Extract final model

final_gbm_model <- best_gbm_models[[as.character(optimal_gbm$VariableCount)]]

final_gbm_vars <- strsplit(optimal_gbm$Variables, ", ")[[1]]

# 5. Final GBM Model Evaluation

cat("\n=== Final GBM Model Evaluation ===\n")

# Predict on training set

gbm_pred <- predict(final_gbm_model, newdata = gbm_data[, c(final_gbm_vars, "survival_class")], type = "prob")

rt$gbm_risk_score <- gbm_pred[, "短期生存"]

# Calculate AUC

gbm_roc <- roc(response = ifelse(rt$survival_class == "短期生存", 1, 0),

predictor = rt$gbm_risk_score)

final_gbm_auc <- auc(gbm_roc)

cat("Final GBM model AUC:", round(final_gbm_auc, 4), "\n")

# Save GBM performance comparison results

write.csv(gbm_performance, file.path(results_dir, "GBM_performance_comparison_different_variables.csv"), row.names = FALSE)

# 6. XGBoost Model - Pursuing Optimal Cost-Effectiveness

cat("\n=== XGBoost Model - Pursuing Optimal Cost-Effectiveness ===\n")

# Prepare XGBoost input data

xgb_data <- rt[, c(predictor_names, "survival_class")]

xgb_data$survival_class <- as.factor(xgb_data$survival_class)

# Convert categorical variable to numeric (XGBoost requires numeric input)

xgb_data$survival_class_numeric <- ifelse(xgb_data$survival_class == "短期生存", 1, 0)

# Create feature matrix and label vector

xgb_features <- as.matrix(xgb_data[, predictor_names])

xgb_label <- xgb_data$survival_class_numeric

# Train initial XGBoost model to obtain feature importance

cat("Training initial XGBoost model to obtain feature importance...\n")

dtrain <- xgb.DMatrix(data = xgb_features, label = xgb_label)

params <- list(

objective = "binary:logistic",

eval_metric = "auc",

max_depth = 3,

eta = 0.1

)

xgb_base <- xgb.train(

params = params,

data = dtrain,

nrounds = 100,

verbose = 0

)

# Get feature importance

xgb_imp <- xgb.importance(model = xgb_base, feature_names = predictor_names)

cat("XGBoost feature importance ranking:\n")

print(xgb_imp)

# Save feature importance

write.csv(xgb_imp, file.path(results_dir, "XGBoost_initial_feature_importance.csv"), row.names = FALSE)

# 7. Identifying Optimal XGBoost Variable Combination

cat("\n=== Identifying Optimal XGBoost Variable Combination ===\n")

# Set up cross-validation

ctrl_xgb <- trainControl(

method = "cv",

number = 5,

classProbs = TRUE,

summaryFunction = twoClassSummary,

savePredictions = "final"

)

# Set up parameter grid (simplified version)

xgbGrid <- expand.grid(

nrounds = c(50, 100), # Number of iterations

max_depth = c(3, 6), # Maximum depth of trees

eta = c(0.01, 0.1), # Learning rate

gamma = 0, # Minimum loss reduction

colsample_bytree = 0.8, # Column sampling ratio per tree

min_child_weight = 1, # Minimum sum of instance weight needed in child

subsample = 0.8 # Row sampling ratio per tree

)

# Test different numbers of important variables

xgb_performance <- data.frame()

best_xgb_models <- list()

max_vars <- min(6, nrow(xgb_imp)) # Test up to 6 variables

for (n_vars in 1:max_vars) {

# Select the top n_vars most important variables

current_vars <- xgb_imp$Feature[1:n_vars]

cat("Testing", n_vars, "variables:", paste(current_vars, collapse = ", "), "\n")

# Prepare data subset - ensure column names are preserved

current_features <- as.data.frame(xgb_features[, current_vars])

colnames(current_features) <- current_vars # Add column names

# Train XGBoost model

xgb_model <- train(

x = current_features,

y = make.names(xgb_data$survival_class),

method = "xgbTree",

trControl = ctrl_xgb,

tuneGrid = xgbGrid,

metric = "ROC",

verbose = FALSE

)

# Get best parameters

best_tune <- xgb_model$bestTune

cat("Best parameters: nrounds=", best_tune$nrounds,

"max_depth=", best_tune$max_depth,

"eta=", best_tune$eta, "\n")

# Predict on training set

xgb_pred <- predict(xgb_model, newdata = current_features, type = "prob")

current_auc <- xgb_model$results %>%

filter(nrounds == best_tune$nrounds,

max_depth == best_tune$max_depth,

eta == best_tune$eta) %>%

pull(ROC)

cat("Cross-validated AUC:", round(current_auc, 4), "\n")

# Record results

xgb_performance <- rbind(xgb_performance,

data.frame(

VariableCount = n_vars,

AUC = current_auc,

Variables = paste(current_vars, collapse = ", "),

nrounds = best_tune$nrounds,

max_depth = best_tune$max_depth,

eta = best_tune$eta

))

# Save best model

best_xgb_models[[as.character(n_vars)]] <- xgb_model

}

# 8. Select Optimal XGBoost Cost-Effectiveness Model

cat("\n=== Select Optimal XGBoost Cost-Effectiveness Model ===\n")

# Calculate cost-effectiveness metric (AUC / number of variables)

xgb_performance$Efficiency <- xgb_performance$AUC / xgb_performance$VariableCount

# Set performance threshold (acceptable AUC drop)

performance_threshold <- 0.03

max_auc <- max(xgb_performance$AUC)

# Find the minimum variable set meeting performance requirements

viable_xgb <- xgb_performance %>%

filter(AUC >= max_auc - performance_threshold) %>%

arrange(VariableCount)

if (nrow(viable_xgb) > 0) {

# Select the model with the fewest variables

optimal_xgb <- viable_xgb[1, ]

} else {

# If none meet the condition, select the model with the best cost-effectiveness

optimal_xgb <- xgb_performance[which.max(xgb_performance$Efficiency), ]

}

cat("\n★ Optimal XGBoost Cost-Effectiveness Model Configuration:\n")

cat("Number of variables:", optimal_xgb$VariableCount, "\n")

cat("Variables:", optimal_xgb$Variables, "\n")

cat("AUC:", round(optimal_xgb$AUC, 4), "\n")

cat("Cost-effectiveness metric:", round(optimal_xgb$Efficiency, 4), "\n")

# Extract final model

final_xgb_model <- best_xgb_models[[as.character(optimal_xgb$VariableCount)]]

final_xgb_vars <- strsplit(optimal_xgb$Variables, ", ")[[1]]

# 9. Final XGBoost Model Evaluation

cat("\n=== Final XGBoost Model Evaluation ===\n")

# Predict on training set

xgb_pred <- predict(final_xgb_model, newdata = as.data.frame(xgb_features[, final_xgb_vars]), type = "prob")

# Check prediction result column names

cat("Prediction result column names:", colnames(xgb_pred), "\n")

# Ensure correct column names are used

if ("X短期生存" %in% colnames(xgb_pred)) {

rt$xgb_risk_score <- xgb_pred[, "X短期生存"]

} else if ("Xshort" %in% colnames(xgb_pred)) {

rt$xgb_risk_score <- xgb_pred[, "Xshort"]

} else if (ncol(xgb_pred) == 2) {

# Use second column (usually positive class)

rt$xgb_risk_score <- xgb_pred[, 2]

} else {

# Use first column

rt$xgb_risk_score <- xgb_pred[, 1]

}

# Calculate AUC

xgb_roc <- roc(response = ifelse(rt$survival_class == "短期生存", 1, 0),

predictor = rt$xgb_risk_score)

final_xgb_auc <- auc(xgb_roc)

cat("Final XGBoost model AUC:", round(final_xgb_auc, 4), "\n")

# 10. Model Performance Comparison Visualization

cat("\n=== Model Performance Comparison Visualization ===\n")

# Standardize column names

colnames(gbm_performance) <- c("VariableCount", "AUC", "Variables", "nrounds", "max_depth", "eta", "Efficiency")

colnames(xgb_performance) <- c("VariableCount", "AUC", "Variables", "nrounds", "max_depth", "eta", "Efficiency")

# Combine GBM and XGBoost results

all_performance <- rbind(

gbm_performance %>% mutate(Model = "GBM"),

xgb_performance %>% mutate(Model = "XGBoost")

)

# Plot relationship between variable count and AUC

perf_plot <- ggplot(all_performance, aes(x = VariableCount, y = AUC, color = Model)) +

geom_line(size = 1.5) +

geom_point(size = 3) +

geom_vline(data = all_performance %>%

filter((Model == "GBM" & VariableCount == optimal_gbm$VariableCount) |

(Model == "XGBoost" & VariableCount == optimal_xgb$VariableCount)),

aes(xintercept = VariableCount, color = Model),

linetype = "dashed", size = 1) +

labs(title = "Model Performance vs Number of Variables",

subtitle = paste("GBM optimal:", optimal_gbm$VariableCount, "variables, AUC=", round(optimal_gbm$AUC, 4),

"\nXGBoost optimal:", optimal_xgb$VariableCount, "variables, AUC=", round(optimal_xgb$AUC, 4)),

x = "Number of Variables", y = "AUC") +

theme_minimal() +

scale_color_manual(values = c("#2E9FDF", "#E7B800"))

# Save performance comparison plot

perf_file <- file.path(results_dir, "GBM_XGBoost_model_performance_comparison.png")

png(perf_file, width = 3000, height = 2000, res = 300)

print(perf_plot)

dev.off()

# 11. Final Model Validation

cat("\n=== Final Model Validation ===\n")

# Function: Bootstrap validation

bootstrap_validate <- function(model, data, vars, model_type, n_boot = 50) {

boot_auc <- numeric(n_boot)

success_count <- 0

for (i in 1:n_boot) {

cat(model_type, "Bootstrap iteration:", i, "/", n_boot, "\n")

tryCatch({

# Resampling with replacement

boot_sample_idx <- sample(nrow(data), replace = TRUE)

boot_sample <- data[boot_sample_idx, ]

# Prepare bootstrap sample data

boot_data <- boot_sample[, c(vars, "survival_class")]

# Train model

if (model_type == "GBM") {

boot_model <- train(

survival_class ~ .,

data = boot_data,

method = "gbm",

trControl = trainControl(method = "none", classProbs = TRUE),

tuneGrid = model$bestTune,

metric = "ROC",

verbose = FALSE

)

# Predict on original data

boot_pred <- predict(boot_model, newdata = data[, c(vars, "survival_class")], type = "prob")

boot_risk_score <- boot_pred[, "短期生存"]

} else { # XGBoost

boot_features <- as.matrix(boot_data[, vars])

boot_model <- train(

x = boot_features,

y = make.names(boot_data$survival_class),

method = "xgbTree",

trControl = trainControl(method = "none", classProbs = TRUE),

tuneGrid = model$bestTune,

metric = "ROC",

verbose = FALSE

)

# Predict on original data

boot_pred <- predict(boot_model, newdata = as.matrix(data[, vars]), type = "prob")

boot_risk_score <- boot_pred[, "X短期生存"]

}

# Calculate AUC

boot_roc <- roc(response = ifelse(data$survival_class == "短期生存", 1, 0),

predictor = boot_risk_score)

boot_auc[i] <- auc(boot_roc)

success_count <- success_count + 1

cat(" Completed successfully, AUC:", round(boot_auc[i], 4), "\n")

}, error = function(e) {

cat(" Iteration failed:", e$message, "\n")

boot_auc[i] <<- NA

})

}

# Calculate statistics

successful_boot <- !is.na(boot_auc)

if (sum(successful_boot) > 0) {

boot_mean <- mean(boot_auc[successful_boot], na.rm = TRUE)

boot_sd <- sd(boot_auc[successful_boot], na.rm = TRUE)

boot_ci <- quantile(boot_auc[successful_boot], c(0.025, 0.975), na.rm = TRUE)

} else {

boot_mean <- boot_sd <- NA

boot_ci <- c(NA, NA)

}

return(list(

boot_auc = boot_auc,

boot_mean = boot_mean,

boot_sd = boot_sd,

boot_ci = boot_ci,

success_count = success_count

))

}

# GBM model bootstrap validation

cat("\n=== GBM Model Bootstrap Validation ===\n")

gbm_boot <- bootstrap_validate(final_gbm_model, rt, final_gbm_vars, "GBM", n_boot = 50)

# XGBoost model bootstrap validation

cat("\n=== XGBoost Model Bootstrap Validation ===\n")

xgb_boot <- bootstrap_validate(final_xgb_model, rt, final_xgb_vars, "XGBoost", n_boot = 50)
